# Health state utilities associated with attributes of migraine preventive treatments based on patient and general population preferences

**DOI:** 10.1007/s11136-019-02163-3

**Published:** 2019-03-28

**Authors:** Louis S. Matza, Kristen A. Deger, Pamela Vo, Farooq Maniyar, Peter J. Goadsby

**Affiliations:** 1 Patient-Centered Research, Evidera, 7101 Wisconsin Avenue, Suite 1400, Bethesda, MD 20814 USA; 2 Modeling and Simulation, Evidera, Bethesda, MD USA; 3 Novartis Pharma AG, Basel, Switzerland; 4 Basildon and Thurrock University Hospitals and Queen Mary University, London, UK; 5 NIHR-Wellcome Trust King’s Clinical Research Facility, King’s College London, London, UK

**Keywords:** Migraine, Utility, Route of administration, Adverse events, Time trade-off, Treatment process utilities

## Abstract

**Purpose:**

While previous studies have estimated health state utilities associated with migraine severity and frequency, migraine treatments vary in other ways that may have an impact on patients’ quality of life, preference, and utility. The purpose of this study was to estimate utilities associated with migraine treatment attributes including route of administration and treatment-related adverse events (AEs).

**Methods:**

In time trade-off interviews, migraine patients and general population participants in the UK valued health state vignettes drafted based on literature, medication labels, and clinician interviews. All respondents valued migraine health states varying in route of administration. Each participant also valued eight health states (randomly selected from a total of 15) that added the description of an AE to a migraine health state.

**Results:**

A total of 400 participants completed interviews (200 general population [49.0% female; mean age = 43.6 years]; 200 migraine patients [74.5% female; mean age = 45.8 years]). In the general population sample, mean utilities of health states without aura were 0.79 with daily oral medication, 0.78 with one injection per month, and 0.72 with 31–39 injections once every 3 months. The greatest disutilities (i.e., decreases in utility) were for AEs associated with oral medications (e.g., − 0.060 [fatigue] and − 0.098 [brain fog]). Differences among health states followed the same pattern in the patient sample as in the general population sample.

**Conclusions:**

Utilities estimated from the general population sample may be used to represent route of administration and AEs in cost-utility models. Results from the patient sample indicate that these treatment characteristics have an impact on patient preference.

## Introduction

Cost-utility analyses (CUAs) are conducted to examine the value of migraine preventive therapies to inform decision-making on healthcare resource allocation [1–6]. To calculate quality-adjusted life years (QALYs), these economic models require utilities, which are values representing the strength of preference for health states on a scale anchored to 0 (dead) and 1 (full health) [7]. Utilities are available to represent levels of migraine severity defined in terms of headache frequency or pain severity [1, 3, 4, 8–13]. However, little is known about the utility impact of other aspects of migraine preventive therapies that could be important to patients, such as route of administration and adverse events (AEs).

Recently, a new class of monoclonal antibodies targeting the calcitonin gene-related peptide (CGRP) or its receptor have demonstrated efficacy in the preventative treatment of episodic and chronic migraine [14–17]. These medications have a different route of administration than previously available treatments, and these differences could impact treatment preferences. Three of the four CGRP-targeted monoclonal antibody treatments are generally administered as a monthly injection, including erenumab [18–20], fremanezumab [21, 22], and galcanezumab [23, 24]. In contrast, the most commonly used migraine preventive therapies are daily oral medications, including topiramate, beta blockers such as propranolol, and tricyclic antidepressants such as amitriptyline [25]. Another treatment that has demonstrated efficacy for chronic migraine is onabotulinumtoxinA, administered every 12 weeks in a series of 31–39 intramuscular injections to the head and neck [26, 27]. No published utilities were located to represent these treatment process differences in CUAs of migraine preventive treatments.

These three medication groups also differ in tolerability. Among the oral migraine preventives, common AEs include paresthesia, somnolence, dizziness, fatigue, and difficulty concentrating [25]. Studies of onabotulinumtoxinA have found the most common AEs to be neck pain and muscular weakness [26, 27]. Clinical trials of the CGRP-targeted medications generally report relatively mild AEs including injection site pain and pruritus (i.e., itching) [15, 16]. No previous studies were located that identified disutilities (i.e., decreases in utility) for AEs associated with these treatments.

These differences among migraine preventive treatments could have an impact on quality of life, health state preference, and utility. Therefore, the purpose of this study was to estimate utilities associated with route of administration and AEs of migraine preventive treatments so that these values could be used in CUAs. The sample included general population participants and migraine patients in the UK. The general population sample was included because Health Technology Assessment (HTA) agencies often prefer utilities based on general population preferences [28]. The patient sample was included to examine patient preference among the treatment attributes, as well as the extent to which the general population values and patient values followed similar patterns.

Generic preference-based measures such as the EQ-5D and Health Utilities Index are useful for estimating utilities associated with migraine severity [8, 13], but these instruments were not designed to be sensitive to specific treatment attributes. Route of administration could be important to patients and have an impact on treatment preference and quality of life. However, preferences associated with route of administration may not be detected by generic instruments, which include items focusing on global health concepts rather than specific aspects of healthcare. The utility impact of AEs is also difficult to estimate with generic instruments for several reasons. First, scores on the generic instruments are influenced by all aspects of health, and it is difficult to know whether a score decrease can be specifically attributed to an AE. Second, many AEs are temporary or intermittent, and it is not always feasible to administer a generic instrument to patients at the time an AE occurs. Third, while some AEs may influence a response to a global item (e.g., injection site pain may be captured by the EQ-5D pain item), other AEs may not have an impact on any item of a generic instrument. For instance, it is possible that paresthesia, brain fog, and fatigue could be bothersome to patients but not have an impact on EQ-5D responses. Thus, like most other studies estimating treatment process utilities [29], this study used a vignette-based approach, which is well suited for isolating the utility impact of specific treatment characteristics.

## Methods

### Overview of study design

Health states (i.e., vignettes) were developed and refined based on published literature, clinician interviews, and a pilot study. All health states described migraine with or without aura (described in the health states as a visual disturbance preceding headaches), receiving treatment via one of three routes of administration. Additional health states added AEs to the health states describing migraine without aura.

Utilities were elicited in a time trade-off (TTO) task with a 10-year time horizon [7]. The interviews were conducted in May and December 2017 in Edinburgh and London, UK. Participants provided written informed consent, and procedures were approved by an independent Institutional Review Board (Ethical & Independent Review Services; Study Number 17035). General population respondents were compensated 40 British pounds. Patients received a larger remuneration (60 pounds) because they completed additional forms to report symptoms and other aspects of migraine history.

### Health state development

A targeted literature search was conducted to ensure that health state descriptions were consistent with published research and to inform development of the interview guide used with the clinicians. The literature review focused on route of administration for commonly used or currently investigated migraine preventive treatments [15, 18–27] and AEs associated with these treatments [15, 16, 26, 30–33].

Multiple rounds of telephone interviews were conducted with two neurologists specializing in headache in the UK. Each neurologist participated in multiple discussions so they could respond to drafts of the health states as they developed. Initial questions for the physicians focused on patients’ typical experience with migraine while on treatment (including route of administration and AEs). The treatments described in the health states are prescribed for both chronic (15 or more migraine days per month) and episodic (less than 15 migraine days per month) migraine. Thus, the migraine episode frequency in the health states was intended to be a plausible representation of either chronic or episodic migraine patients whose migraine frequency has decreased with treatment. The clinicians helped identify a typical range of migraine days per month that may be experienced by either a chronic or episodic migraine sufferer when successfully treated. For these health states, all three treatments were assumed to have equal efficacy. After the health states were drafted, discussions with the clinicians focused on reviewing and editing drafts to ensure they were clear and accurate representations of the typical patient experience.

A total of 21 health states were drafted, including two groups of treatment process health states (group 1 without aura and group 2 with aura) and 15 AE health states. Previous research has focused on advantages and disadvantages of naming a medical condition in health state vignettes [34–37]. In this study, the term “migraine” was used because many respondents would likely identify this condition based on the health state content. Therefore, it could have been confusing if the term “migraine” was not used. Furthermore, migraine is not typically associated with stigma like some other medical conditions, and therefore, naming the condition has less potential to introduce bias.

The health states were presented to respondents on individual cards with bullet point descriptions. For the treatment process health states (A–F), the bullet points were organized into categories with headings including “Description,” “Treatment,” “Symptoms,” and “Impact.” Health states D–F added a section describing aura (“Visual Disturbance”). AE health states (G–U) were written as brief bullet points to be added to health states A, B, or C. See “Appendix” for health state text.

Group 1 health states (A, B, C) described migraine without aura, including symptoms, migraine frequency, impact on functioning, and treatment modality. These three health states were identical except for route of treatment administration. Health state A described a once-monthly injection, which is the most common administration mode of the CGRP-targeted therapies [15]. Health state B described 31–39 injections once every 3 months, representing onabotulinumtoxinA [26, 27]. Health state C described oral treatment with tablets taken one to two times daily, consistent with treatments such as propranolol, topiramate, and amitriptyline [25]. Because everything about these three health states was held constant, except for route of administration, any difference in preference could be attributed to this difference in treatment process. Group 2 health states (D, E, F) were identical to group 1 except for the addition of migraine aura.

The 15 AE health states described AEs associated with CGRP-targeted therapies (group 3), onabotulinumtoxinA (group 4), and oral treatments (group 5). These AEs were selected because they were frequently reported in clinical trials of these treatments and considered to be common by the two neurologists consulted for this study. Group 3 health states represented AEs associated with CGRP-targeted therapies: G (injection site pain) [15], H (respiratory tract infection), and I (pruritus) [15]. Respiratory tract infection has been reported in trials across all classes of medications in this study as well as in placebo comparator groups [15, 31, 38–40]. It is included in the group 3 health states because it was added to health state A for the assessment of disutility, but it is similarly relevant to all these medication classes.

Group 4 health states describing AEs associated with onabotulinumtoxinA included J, K, and L, representing neck stiffness and pain [32], eyelids/eyebrow drooping [32], and muscle weakness [32], respectively. Group 5 described AEs associated with oral preventive therapies. These health states included M (fatigue) [30, 31], N (exercise intolerance) [33], O (dizziness) [31], P (insomnia) [30], Q (paresthesia) [30, 31], R (brain fog) [30, 31], S (drowsiness) [30, 31], T (dry mouth) [31], and U (constipation) [31].

### Participants

General population participants and patients diagnosed with migraines were required to be at least 18 years old; able to understand the assessment procedures; willing to provide informed consent; and a UK resident. The migraine patients were also required to have been diagnosed with migraine, received prescription medication for migraine, and able to provide proof of diagnosis or treatment (e.g., documentation of diagnosis from a healthcare provider, proof of prescription medication for migraine). Participants were recruited via newspapers and online advertisements.

### Pilot study

To assess clarity of health states and methods, a pilot study was conducted in London with 11 general population participants (36.4% male; mean age = 34.2 years) and four migraine patients (75.0% male; mean age = 46.0 years). The participants valued the health states in a TTO task. Some participants found it difficult to remember differences among the large number of AEs, and therefore, it was decided to decrease the number of AEs presented to each participant. It was determined that a subset of eight AEs would be feasible for most respondents. Participants generally reported that the health state language and concepts were clear and comprehensible. Some participants suggested revisions in formatting and word choice, and the health states were edited accordingly.

### Utility interview procedures and scoring

After finalizing health states and methods in the pilot study, health state utilities were elicited in a TTO valuation study. All participants began by ranking the six treatment process health states (A to F, presented in random order) from most preferable to least preferable as an introductory task to help them learn the health state content, followed by valuation of these states in the TTO task. Then, participants were randomly assigned to rank and value eight of the 15 AE health states (G–U). Each adverse event was added to either health state A, B, or C, depending on the treatment with which the adverse event is typically associated.

The TTO valuation was conducted with a 10-year time horizon and 6-month (i.e., 5%) trading increments. For each health state, participants were offered a choice between living 10 years in the health state being rated or a shorter duration in full health. TTO choices were presented in an order alternating between longer and shorter durations in full health: 10 years, 0 (i.e., dead), 9.5, 0.5, 9, 1, 8.5, 1.5, 8, 2, etc. For health states perceived as better than dead, utilities (u) were calculated based on the point of equivalence between the two choices as the number of years in full health (x) divided by the number of years in the health state being rated (u = x/10 years).

In rare circumstances when participants perceived a health state to be worse than dead, the task and scoring procedures were adjusted [41]. Participants were given the choice between dead (choice 1) and a 10-year life span (choice 2) beginning with varying amounts of time in the health state being rated, followed by full health for the rest of the 10 years. The resulting negative utility scores were calculated based on the point of equivalence with a bounded scoring approach commonly used to avoid highly skewed distributions for negative utility scores (u = − x/10, where x is the number of years in full health, and 10 is the number of years in the total life span of choice 2).

### Demographic and clinical measures

All participants completed a brief demographic and clinical information form to report age, gender, marital status, employment, education level, racial/ethnic background, and presence of health conditions. Migraine patients also completed the questionnaire focusing on their migraine diagnosis, symptoms, and treatment.

### Statistical analysis procedures

Statistical analyses were completed using SAS version 9.4. Continuous variables are summarized as means and standard deviations and categorical demographic variables as frequencies and percentages. Disutility (i.e., decrease in utility) associated with AEs was calculated as the difference between otherwise identical health states with and without an AE. For example, the difference between health states B and J is the disutility of neck stiffness and pain in the context of treatment with 31–39 injections once every 3 months. Student’s *t* tests were used to compare utilities between subgroups with the Bonferroni adjustment for multiple comparisons between general population respondents and migraine patients (e.g., for a table with 15 comparisons, the *p* value threshold of 0.05 would be divided by 15 so that *p* values < 0.003 would be considered statistically significant). Comparisons between utilities of various health states were examined with paired *t* tests and analyses of variance (ANOVAs) with Bonferroni-adjusted post hoc pairwise comparisons.

## Results

### General population sample description

A total of 238 potential general population participants were scheduled for interviews, 207 attended, and seven had difficulty understanding the health states and/or TTO procedures. Thus, 200 valid TTO interviews were conducted (107 Edinburgh; 93 London). See demographics in Table 1. Commonly reported health conditions were anxiety (18.5%), depression (15.5%), and sleep problems (13.0%). In addition, 29 (14.6%) of the general population participants reported being diagnosed with migraines, which is consistent with epidemiological research. Migraine has been estimated to occur in about 15% of the UK adult population [42, 43] and 15% globally [44, 45].

**Table 1:** Demographic characteristics

	General population sample (*N* = 200)	Migraine patient sample (*N* = 200)
Age (mean, SD)	43.6 (16.2)	45.8 (15.2)
Gender (*n*, %)		
Male	102 (51.0%)	51 (25.5%)
Female	98 (49.0%)	149 (74.5%)
Ethnic/racial background (*n*, %)		
White	155 (77.5%)	154 (77.0%)
Asian	19 (9.5%)	12 (6.0%)
Black	15 (7.5%)	20 (10.0%)
Mixed^a^	7 (3.5%)	12 (6.0%)
Other^b,c^	4 (2.0%)	2 (1.0%)
Marital status (*n*, %)		
Not married^d^	105 (52.5%)	119 (59.5%)
Married/cohabitating/living with partner	95 (47.5%)	81 (40.5%)
Employment status (*n*, %)		
Full-time work	80 (40.0%)	83 (41.5%)
Part-time work	54 (27.0%)	44 (22.0%)
Other^e^	66 (33.0%)	73 (36.5%)
Education level (*n*, %)		
University degree	105 (52.5%)	101 (50.5%)
No university degree	95 (47.5%)	99 (49.5%)

^a^Mixed ethnic/racial background includes White and Black Caribbean; White and Black African; White and Asian; and other mixed/multiple ethnic groups as reported by respondents

^b^Other ethnic/racial background in the general population sample includes Arab (1), African/American Indigenous People/Caucasian (1), half UK/Iraqi (1) and Somali (1) as reported by respondents

^c^Other ethnic/racial background in the migraine patient sample includes Arab (2)

^d^Not married includes single, divorced, separated, and widowed

^e^Other employment includes homemaker, student, unemployed, retired, disabled, and other as reported by the respondents

### Migraine patient sample description

Of 248 potential migraine patients who were scheduled, 203 attended interviews. Three had difficulty understanding the health states and/or procedures. Thus, 200 valid TTO interviews were conducted (98 Edinburgh; 102 London). Demographics (Table 1) were similar to those of the general population sample except for the gender distribution, consistent with research suggesting that migraine is more common in women by a three-to-one ratio [42, 44, 46]. The most commonly reported comorbidities among the migraine patients were anxiety (33.5%), depression (28.5%), sleep problems (26.5%), and asthma (12.0%). The majority reported having their most recent migraine within the past week (60.5%). The average number of days per month with migraine symptoms was 7.3, and the average age at the time of the first migraine episode was 21.2 years. Clinical characteristics were similar in London and Edinburgh (Table 2).

**Table 2:** Clinical characteristics of the migraine patient sample

	Edinburgh (*N* = 98)	London (*N* = 102)	Total migraine patient sample (*N* = 200)	*p* value^a^
Diagnosed with migraines (*n*, %)	98 (100.0%)	102 (100.0%)	200 (100.0%)	
Days per month with headache symptoms (mean, SD)	9.2 (7.8)	9.1 (7.9)	9.2 (7.8)	0.97
Days per month with migraine symptoms (mean, SD)	8.7 (7.3)	9.1 (7.4)	8.9 (7.3)	0.66
Time of most recent migraine (*n*, %)				0.49
Within the past 24 h	21 (21.4%)	21 (20.6%)	42 (21.0%)	
Within the past week	32 (32.7%)	47 (46.1%)	79 (39.5%)	
Within the past month	32 (32.7%)	24 (23.5%)	56 (28.0%)	
Within the past 3 months	6 (6.1%)	6 (5.9%)	12 (6.0%)	
Within the past 6 months	5 (5.1%)	3 (2.9%)	8 (4.0%)	
Within the past year	1 (1.0%)	0 (0.0%)	1 (0.5%)	
More than 1 year ago	1 (1.0%)	1 (1.0%)	2 (1.0%)	
Age at first migraine (mean, SD)	20.1 (9.7)	22.3 (12.4)	21.2 (11.2)	0.18

^a^*P* values are based on Chi-square analyses for categorical variables and *t* tests for continuous variables

### Health state utilities

Mean utility scores for each health state were numerically higher in the migraine sample than the general population sample, but the pattern of preferences and differences among health states was almost identical in the two samples (Table 3). Comparisons of health state means between the general population and migraine samples revealed only one *p* value less than 0.05 across all 21 health states (S, drowsiness). After applying the Bonferroni adjustment to the significance threshold in this table (0.05/21 = 0.002), the between-group differences for all health states (including S) are considered nonsignificant.

**Table 3:** Time trade-off (TTO) utility scores

Health states	General population (GP) sample		Migraine patient sample		Comparison of utilities between GP and migraine samples	
	*N*	Mean^a^ (SD)	*N*	Mean^a^ (SD)	*t*	*p* value
Group 1: Migraine without aura						
A. Migraine without aura treated with 1 injection per month	200	0.78 (0.22)	200	0.82 (0.23)	− 1.50	0.13
B. Migraine without aura treated with 31–39 injections per month	200	0.72 (0.23)	200	0.75 (0.28)	− 0.96	0.34
C. Migraine without aura treated with orals	200	0.79 (0.22)	200	0.83 (0.22)	− 1.38	0.17
Group 2: Migraine with aura						
D. Migraine with aura treated with 1 injection per month	200	0.76 (0.23)	200	0.79 (0.25)	− 1.30	0.19
E. Migraine with aura treated with 31–39 injections per 3 months	200	0.69 (0.25)	200	0.73 (0.29)	− 1.46	0.15
F. Migraine with aura treated with orals	200	0.77 (0.23)	200	0.80 (0.23)	− 1.38	0.17
Group 3: Adverse events associated with 1 injection per month (added to health state A)						
G. Injection site pain	106	0.77 (0.26)	109	0.80 (0.25)	− 0.97	0.33
H. Respiratory tract infection^b^	108	0.77 (0.25)	107	0.79 (0.26)	− 0.63	0.53
I. Pruritus	105	0.77 (0.26)	105	0.80 (0.25)	− 0.91	0.36
Group 4: Adverse events associated with 31–39 injections per 3 months (added to health state B)						
J. Neck stiffness and pain	105	0.67 (0.28)	106	0.70 (0.33)	− 0.55	0.58
K. Eyelids/eyebrow drooping	106	0.71 (0.21)	106	0.69 (0.34)	0.54	0.59
L. Muscle weakness	107	0.68 (0.22)	103	0.70 (0.34)	− 0.50	0.62
Group 5: Adverse events associated with oral medications^c^ (added to health state C)						
M. Fatigue	103	0.73 (0.24)	104	0.77 (0.25)	− 1.29	0.20
N. Exercise intolerance	105	0.74 (0.23)	105	0.80 (0.24)	− 1.85	0.07
O. Dizziness	107	0.79 (0.18)	106	0.81 (0.20)	− 0.97	0.33
P. Insomnia	106	0.76 (0.20)	104	0.78 (0.20)	− 0.76	0.45
Q. Paresthesia	106	0.79 (0.19)	104	0.83 (0.20)	− 1.53	0.13
R. Brain fog	104	0.68 (0.28)	106	0.72 (0.30)	− 0.94	0.35
S. Drowsiness	104	0.75 (0.26)	106	0.82 (0.13)	− 2.54	0.01
T. Dry mouth	106	0.78 (0.25)	106	0.81 (0.25)	− 0.85	0.40
U. Constipation	106	0.78 (0.24)	106	0.78 (0.31)	0.04	0.97

^a^TTO scores are on a scale anchored with 0 representing dead and 1 representing full health

^b^Respiratory tract infection has been reported in trials across all classes of medications in this table as well as in placebo comparator groups. It is included in the Group 3 health states here because it was added to health state A for the assessment of disutility, but it is similarly relevant to all these medication classes

^c^Oral medications including beta blockers (e.g., propranolol), tricyclic antidepressants (e.g., amitriptyline), and antiepileptic treatments (e.g., topiramate)

Almost all participants perceived all health states to be better than dead, resulting in positive utility scores. Among general population participants, the group 1 (A–C) and group 2 (D–F) states were rated worse than dead by 1.0% and 1.5% of the sample, respectively. The adverse event health states had similarly low rates of negative utilities. Rates of negative scores in the migraine sample were in a similar low range for most health states, but slightly higher for some AE states (e.g., L was rated worse than dead by 3.9% of the migraine patient sample).

### Utility differences associated with route of administration

Among route of administration health states, C (migraine without aura treated with orals) had the highest mean utility in both samples, followed by A, F, D, B, and E (Table 3). The differences in utility values for health states without aura vs. the corresponding health states with aura (A vs. D; B vs. E; C vs. F) were always statistically significant (*p* < 0.0001). Four ANOVAs were conducted to examine differences associated with route of administration (Tables 4a and 4b). The overall F values of all ANOVAs were statistically significant. In the general population sample, post hoc pairwise comparisons with the Bonferroni adjustment found significant differences between utilities of health states representing 31–39 injections every 3 months and utilities of health states describing the other two routes of administration. However, no significant differences in utility were found between oral treatment and one injection per month. For example, the utility of health state B was significantly different from A and C, but there was no significant difference between A and C. Results followed a similar pattern in the migraine sample.

**Table 4a:** Health state utilities associated with route of administration: Migraine without aura

	Mean (SD) utilities of health states describing migraine without Aura			ANOVA comparing the three utilities		Pairwise comparisons: Mean (SD) difference scores^a^		
	A. 1 injection per month	B. 31–39 injections once every 3 months	C. Oral treatment	*F*	Overall *p* value	A vs. B	A vs. C	B vs. C
General population	0.783 (0.221)	0.724 (0.233)	0.795 (0.220)	5.75	0.0034	0.059* (0.084)	− 0.012 (0.041)	− 0.071** (0.096)
Migraine patients	0.817 (0.230)	0.748 (0.276)	0.826 (0.225)	5.98	0.0027	0.069* (0.138)	− 0.009 (0.052)	− 0.077** (0.154)

*p < 0.05, **p < 0.01

^a^Post hoc pairwise comparisons with Bonferroni adjustment for multiple comparisons

**Table 4b:** Health state utilities associated with route of administration: Migraine with aura

	Mean (SD) utilities of health states describing migraine with Aura			ANOVA comparing the three utilities		Pairwise comparisons Mean (SD) difference scores^a^		
	D. 1 injection per month	E. 31–39 injections once every 3 months	F. Oral treatment	*F*	Overall *p* value	D vs. E	D vs. F	E vs. F
General population	0.756 (0.230)	0.689 (0.249)	0.766 (0.231)	6.18	0.0022	0.066* (0.102)	− 0.011 (0.044)	− 0.077** (0.108)
Migraine patients	0.787 (0.248)	0.729 (0.287)	0.798 (0.235)	4.21	0.0153	0.058 (0.134)	− 0.011 (0.071)	− 0.070* (0.156)

*p < 0.05, **p < 0.01

^a^Post hoc pairwise comparisons with Bonferroni adjustment for multiple comparisons

### Disutility of adverse events

Disutility scores were calculated for each AE (Table 5). These scores represent the decrease in utility associated with adding each AE to a migraine health state. Among general population participants, disutilities of AEs associated with the CGRP-targeted treatments ranged from − 0.007 (I) to − 0.012 (H). Disutilities of AEs associated with onabotulinumtoxinA ranged from − 0.024 (K) to − 0.045 (J). Disutilities of AEs associated with orals had a wider range from − 0.010 (O) to − 0.098 (R). Of the 15 adverse event health states, the greatest disutilities were for M (fatigue) and R (brain fog).

**Table 5:** Disutilities of adverse events commonly associated with migraine preventive treatments

Disutilities of adverse events^a^	General population (GP) sample		Migraine patient sample		Comparison of disutilities between GP and migraine samples	
	*N*	Mean^a^ (SD)	*N*	Mean^a^ (SD)	t	*p* value
Adverse events associated with CGRP-targeted treatments						
G—A. Injection site pain	106	− 0.009 (0.024)	109	− 0.005 (0.020)	− 1.377	0.17
H—A. Respiratory tract infection^b^	108	− 0.012 (0.034)	107	− 0.015 (0.033)	0.487	0.63
I—A. Pruritus	105	− 0.007 (0.022)	105	− 0.001 (0.009)	− 2.467	0.01
Adverse events associated with OnabotulinumtoxinA						
J—B. Neck stiffness and pain	105	− 0.045 (0.077)	106	− 0.031 (0.109)	− 1.104	0.27
K—B. Eyelids/eyebrow drooping	106	− 0.024 (0.067)	106	− 0.035 (0.115)	0.875	0.38
L—B. Muscle weakness	107	− 0.035 (0.057)	103	− 0.033 (0.123)	− 0.099	0.92
Adverse events associated with oral treatments^c^						
M—C. Fatigue	103	− 0.060 (0.098)	104	− 0.069 (0.144)	0.501	0.62
N—C. Exercise intolerance	105	− 0.047 (0.092)	105	− 0.043 (0.133)	− 0.286	0.78
O—C. Dizziness	107	− 0.010 (0.041)	106	− 0.021 (0.084)	1.185	0.24
P—C. Insomnia	106	− 0.047 (0.087)	104	− 0.063 (0.100)	1.190	0.24
Q—C. Paresthesia	106	− 0.012 (0.045)	104	− 0.013 (0.045)	0.192	0.85
R—C. Brain Fog	104	− 0.098 (0.130)	106	− 0.132 (0.236)	1.268	0.21
S—C. Drowsiness	104	− 0.031 (0.059)	106	− 0.028 (0.057)	− 0.337	0.74
T—C. Dry mouth	106	− 0.011 (0.044)	106	− 0.007 (0.029)	− 0.737	0.46
U—C. Constipation	106	− 0.030 (0.060)	106	− 0.030 (0.139)	0.032	0.97

^a^TTO scores are on a scale anchored with 0 representing dead and 1 representing full health. Disutilities of each adverse event were calculated by subtracting the utility of a health state without any adverse events from the utility of an otherwise identical health state with the addition of a single adverse event

^b^Respiratory tract infection has been reported in trials across all classes of medications in this table as well as in placebo comparator groups. It is included in the Group 3 health states here because it was added to health state A for the assessment of disutility, but it is similarly relevant to all these medication classes

^c^Oral medications including beta blockers (e.g., propranolol), tricyclic antidepressants (e.g., amitriptyline), and antiepileptic treatments (e.g., topiramate)

*CGRP* calcitonin gene-related peptide, *GP* general population, *SD* standard deviation

AE disutilities were similar in the migraine patient sample. Comparisons of disutilities between the general population and migraine samples revealed only one *p* value less than 0.05 across all 15 AE health states (I, pruritis). This disutility was similarly small in both groups (− 0.007 and − 0.001). After applying the Bonferroni adjustment to the significance threshold in this table (0.05/15 = 0.003), the between-group differences for all health states (including I) are considered nonsignificant.

Paired *t* tests comparing utilities of heath state pairs that were identical except for the addition of an AE (e.g., comparing health state A to G, H, and I) found that most AE disutilities were statistically significant. To interpret these *t* tests, statistical significance was set at *p* < 0.003 because there were 15 adverse events (calculated as 0.05/15 following the Bonferroni correction). In the general population sample, significant differences were found for all AEs except those in health states O (dizziness) and T (dry mouth). In the migraine sample, significant differences were found for all AEs except G (injection site pain once per month), I (pruritis), L (muscle weakness), O (dizziness), T (dry mouth), and U (constipation).

### Subgroup comparisons

There were no statistically significant differences in utility or disutility by gender or geographic location (i.e., London vs. Edinburgh) in either the general population or migraine samples (all *p* > 0.05). When dividing the sample into older and younger age groups (by median split), no significant differences were found for the general population sample. In the migraine sample, the older group had slightly greater utility scores only for health states B (*p* = 0.04) and E (*p* = 0.03), but these would not be considered statistically significant after adjusting for multiple comparisons.

Within the migraine patient sample, utility and disutility scores were also compared between subgroups whose frequency of self-reported migraine episodes was consistent with either chronic (15 or more migraine days/month; *n* = 43) or episodic (0–14 days/month; *n* = 157) migraine. No significant differences in utility or disutility were found between these groups.

## Discussion

This is the first study to provide health state utilities that differentiate among migraine preventive therapies in terms of treatment process. The utilities derived from the general population sample may be used to adjust utility scores in models comparing treatments that differ with regard to route of administration or AE profile. The utilities from the migraine patient sample indicate that these treatment characteristics also have a quantifiable effect on patient preference. The utility differences associated with both route of administration and AEs were remarkably similar in the patient and general population samples.

Among health states differing in route of administration, daily oral medication was associated with the highest mean utility score. During the interviews, respondents often noted the ease and familiarity of oral treatment regimens (e.g., “Orals would be easier,” “I’ve been taking oral tablets every day for 5 years”). However, the mean utilities of the monthly injection health states were only slightly lower than the mean utilities of the oral administration health states. While some respondents were averse to self-injections (e.g., “I wouldn’t give myself an injection,” “I’m terrified of needles”), others perceived the monthly injection to be a convenient alternative to daily tablets (e.g., “I like the idea of it being one every 4 weeks rather than popping pills every day”). In contrast, the larger utility decrease associated with the onabatulinumtoxinA treatment process reflects respondents’ adverse reactions to the greater number of injections (e.g., “31–39 injections sound horrible,” “the thought of having all those injections is just something I would not want to deal with ever”).

Mean disutilities varied widely across the 15 AEs. AEs associated with the CGRP-targeted monoclonal antibody treatments were perceived as mild, resulting in small disutilities (− 0.007 to − 0.012 in the general population sample), while disutilities associated with onabotulinumtoxinA were somewhat larger (− 0.024 to − 0.045). Disutilities of AEs associated with oral migraine preventives were highly variable. For example, paresthesia, which is the most common AE with topiramate [30, 31], had a minimal impact on utility. In contrast, other AEs associated with oral medications such as fatigue and brain fog had substantially larger disutilities. Given this variable and possibly large impact on utility, it seems useful to include these differences in CUAs comparing treatments that vary by AE profile.

Researchers developing CUAs to compare migraine preventive treatments may use the results of the current study. Rather than using the absolute value of the utility scores (e.g., 0.78 on the 0 to 1 scale), it is recommended to use the difference scores to adjust utilities. For example, a model comparing an oral medication to a once-monthly injection could use EQ-5D utilities from a clinical trial to represent health status, but these EQ-5D values are unlikely to reflect differences in preference associated with route of administration. Therefore, the difference score of 0.012 (from Table 4a) can be used to adjust the EQ-5D utilities (as either an addition to the oral utility or subtraction from the injection utility). Similarly, the AE disutilities (Table 5) can be applied to specific treatment arms in CUAs, weighted by the percentage of patients who experience these AEs in clinical trials. This approach for adjusting utilities to represent treatment process differences, adverse events, or other treatment characteristics is commonly used in published cost-utility models, including models submitted for HTA review [47–50]. Modelers may also run a CUA first without these adjustments, followed by a sensitivity analysis that includes the utility impact of these treatment characteristics. When adjusting utilities in this way, modelers should be cautious of double counting the disutilities of AEs. Some AEs could have an impact on a utility derived from the EQ-5D or another generic instrument. If so, there would be no need for further utility adjustment to account for the impact of this AE. Researchers should be mindful of this possibility when considering whether to adjust utility scores for specific AEs.

Results add to the literature on treatment process utilities. Although utilities are most often used to quantify health status or treatment outcomes, there is growing interest in patient preferences and health state utilities associated with treatment process attributes [29, 51]. Increasingly, studies are conducted to estimate process utilities for use in economic modeling [35, 52, 53]. Although utility differences associated with the treatment process tend to be relatively small, these treatment process attributes can be important to patients, and they could influence treatment adherence, which has an impact on outcomes [54–56]. Process utilities may be particularly important for differentiating among treatments that have similar efficacy but vary in other ways that affect patients’ quality of life. However, despite the growing number of published studies on process utilities, HTA reviewers may not all agree that treatment process should be included as part of a health state. Therefore, researchers should proceed with appropriate caution when planning to use process utilities in CUAs.

Study limitations should be considered. Most importantly, vignette-based utility estimates are limited by the accuracy and level of detail of the health state descriptions. In this study, the descriptions of route of administration were drafted to be brief, objective, and consistent with drug labels to minimize bias. Similarly, the AE descriptions were carefully drafted in simple language and refined based on published literature and clinician input. Nevertheless, it is not known whether preferences of the current sample would be consistent with preferences of patients who actually had these experiences. Although the patients in the current study had likely experienced many of the symptoms, treatments, and adverse events described in the health states, none of the patients had experienced all relevant concepts. Another study limitation is that results could be influenced by selection bias because the sample consists of participants who were willing and able to respond to newspaper advertisements. The extent to which the study sample is truly representative of the general population or migraine patients is unknown. Therefore, results should be interpreted and used with appropriate consideration of methodological limitations.

Another limitation is that the vignette-based approach is not the preferred utility estimation method in some HTA guidelines. Most notably, the National Institute for Health and Care Excellence (NICE) Guide to the Methods of Technology Appraisal recommends using EQ-5D utilities whenever possible to maximize “consistency across appraisals” [28]. However, the NICE guide also acknowledges that the EQ-5D is not suitable for every situation, and other utility assessment methods may be used when the EQ-5D is not “appropriate.” The current study is an example of a situation where an alternative utility assessment method seemed appropriate. Although generic instruments like the EQ-5D have some items that could possibly be affected by treatment process attributes (e.g., the EQ-5D usual activities item), none of the items specifically target attributes like route of administration. Furthermore, generic instruments cannot isolate the utility impact of a single AE because indices of overall health status would be influenced by symptoms related to the disease as well as the AE.

In contrast, vignette-based methods can isolate the utility impact of specific treatment attributes. By keeping all aspects of health states constant except for a single attribute (e.g., route of administration), it is possible to estimate the utility impact of this attribute (Tables 4a, b, 5). Reviews of treatment process utilities suggest there is an emerging consensus that vignette-based methods are appropriate for this purpose [29, 51]. Still, vignette-based utilities should be used with appropriate caution because comparability to standardized generic-based preference measures is not known.

Despite limitations, this study provides utilities likely to be useful in CUAs comparing the value of migraine preventive therapies. Route of administration and AEs had an impact on preferences of patients and general population respondents, and these preferences resulted in utility differences. These utility differences may be used to more thoroughly represent the effects of these treatments on preference and quality of life when evaluating cost-effectiveness of new treatments.

## Appendix: Health state text

### Health state A: Patient treated for migraine without aura, one injection per month

#### Description

- You have migraine. This is an unpredictable disorder that causes disabling headaches and other associated symptoms.
- You are treated with a medication that reduces the number and duration of migraine episodes.
- Because of the treatment, you have fewer headaches.

#### Treatment

- You are treated with one injection every 4 weeks.
- You give yourself this injection at home.
- You can inject yourself in the thigh or abdomen.

#### Symptoms

- You now have migraine headaches 5 to 8 days each month. Each headache lasts about 2 to 6 h.
- At the beginning of each episode, you feel tired and you notice that you have neck discomfort.
- Each episode includes a very painful headache, usually on one side of your head. The pain is severe and throbbing, and it becomes worse with movement.
- During each episode, it is hard for you to concentrate. You feel like you have “brain fog.”
- During many of these episodes, you feel like you are going to be sick (nausea), and you feel uncomfortably sensitive to light and sound.
- After each migraine episode, you feel tired and worn out.

#### Impact

- On days with a migraine, it is difficult for you to perform everyday tasks.
- For example, when you are affected by migraines, it is difficult to concentrate and be productive at home, work, or school.
- If you have children, it is difficult to care for them on days with a migraine.
- When you have a migraine, you would prefer to stay at home.

### Health state B: Patient treated for migraine without aura, 31–39 injections every 3 months

This health state has the same text as health state A, except for the section titled “Treatment,” which has the following text:

- You are treated with a series of injections once every 3 months.
- At these times, you receive 31–39 injections in your forehead, side of your head, back of your head, and neck.
- The injections are given by a doctor or nurse in their office.

### Health state C: Patient treated for migraine without aura, oral medication

This health state has the same text as health state A, except for the section titled “Treatment,” which has the following text:

- You take one or two tablets once or twice per day.
- You must take these tablets every day.

### Health states D, E, and F: Patient treated for migraine with aura

These three health states have the same text as health states A, B, and C, plus the addition of the following text describing the occurrence of migraine auras with the heading “Visual Disturbance.”

- Before about a third of your headaches, you experience a visual disturbance.
- When this happens, you first see a small spot or sparkle in part of your visual field. The spot grows and causes you to lose part of your vision in both eyes. Some people describe this spot as flashing lights, zig-zagging, or squiggly lines.
- This visual disturbance lasts about 30 min.
- During this experience, you are worried about when your vision will come back.

### Health state G: Injection site pain (the text below was added to health state A)

- As a side effect of your migraine treatment, you have pain at the injection site after each monthly injection.
- You feel sore and have redness in the place where the medication was injected.
- This lasts for about a day.

### Health state H: Upper respiratory tract infection (the text below was added to health state A)

- As a side effect of your migraine treatment, you have symptoms like congestion, cough, and runny nose.
- These symptoms last a few days after each injection.

### Health state I: Pruritus (the text below was added to health state A)

- As a side effect of your migraine treatment, you have itching at the injection site after each monthly injection.
- This lasts for about a day.

### Health state J: Neck stiffness and pain (the text below was added to health state B)

- As a side effect of your migraine treatment, you have pain and stiffness in your head and neck.
- Muscles in your head and neck feel stiff or tight. It is more difficult than usual to move your head for about 2 days after each set of injections.
- You have pain in your neck after you receive the injections. This can last for up to 2 weeks.

### Health state K: Drooping eyelids or eyebrows (the text below was added to health state B)

- As a side effect of your migraine treatment, you notice some drooping of the eyelid on one side of your face.
- This means the eyelid is slightly lower than it usually is.
- This lasts for about a month after each treatment.

### Health state L: Muscle weakness (the text below was added to health state B)

- As a side effect of your migraine treatment, you have muscle weakness in the areas where you receive the injections.
- This primarily affects your neck.
- This lasts for about a month after each treatment.

### Health state M: Fatigue (the text below was added to health state C)

- As a side effect of your migraine treatment, you feel fatigued.
- You feel tired and drained throughout the day.

### Health state N: Exercise intolerance (the text below was added to health state C)

- As a side effect of your migraine treatment, you cannot exercise as vigorously or for as long as you could before you began treatment.
- You feel tired climbing a few flights of stairs.

### Health state O: Dizziness (the text below was added to health state C)

- As a side effect of your migraine treatment, you sometimes feel dizzy when you stand up quickly.

### Health state P: Insomnia (the text below was added to health state C)

- As a side effect of your migraine treatment, you have trouble sleeping.
- It takes you longer to fall asleep and you are more restless when you are sleeping.
- Sometimes you are awoken by nightmares.

### Health state Q: Paresthesia (the text below was added to health state C)

- As a side effect of your migraine treatment, you have a tingling feeling in your hands and feet.
- This feels like “pins and needles,” but it is not painful.

### Health state R: “Brain fog” (the text below was added to health state C)

- As a side effect of your migraine treatment, you have difficulty concentrating.
- You feel like you have “brain fog.”
- You are not able to think as clearly or as quickly as before you began using this medication.
- Sometimes you have trouble finding your words, and you do not remember things as well as before you began using this medication.

### Health state S: Drowsy after waking up (the text below was added to health state C)

- As a side effect of your migraine treatment, you feel very drowsy when you wake up.
- It takes a few hours each day for you to stop feeling drowsy.

### Health state T: Dry mouth (the text below was added to health state C)

- As a side effect of your migraine treatment, your mouth often feels dry.
- This is uncomfortable, and you drink a lot of water to try to make your mouth feel less dry.

### Health state U: Constipation (the text below was added to health state C)

- As a side effect of your migraine treatment, you are often constipated.
